# Simulating Fine-Scale Marine Pollution Plumes for Autonomous Robotic Environmental Monitoring

**DOI:** 10.3389/frobt.2018.00052

**Published:** 2018-05-28

**Authors:** Muhammad Fahad, Yi Guo, Brian Bingham

**Affiliations:** ^1^Robotics and Automation Laboratory, Department of Electrical and Computer Engineering, Stevens Institute of Technology, Hoboken, NJ, United States; ^2^Department of Mechanical and Aerospace Engineering, Naval Postgraduate School, Monterey, CA, United States

**Keywords:** environmental monitoring, pollution plume models, autonomous robotics, plume characteristics, Robot Operating System (ROS) simulator.

## Abstract

Marine plumes exhibit characteristics such as intermittency, sinuous structure, shape and flow field coherency, and a time varying concentration profile. Due to the lack of experimental quantification of these characteristics for marine plumes, existing work often assumes marine plumes exhibit behavior similar to aerial plumes and are commonly modeled by filament based Lagrangian models. Our previous field experiments with Rhodamine dye plumes at Makai Research Pier at Oahu, Hawaii revealed that marine plumes show similar characteristics to aerial plumes qualitatively, but quantitatively they are disparate. Based on the field data collected, this paper presents a calibrated Eulerian plume model that exhibits the qualitative and quantitative characteristics exhibited by experimentally generated marine plumes. We propose a modified model with an intermittent source, and implement it in a Robot Operating System (ROS) based simulator. Concentration time series of stationary sampling points and dynamic sampling points across cross-sections and plume fronts are collected and analyzed for statistical parameters of the simulated plume. These parameters are then compared with statistical parameters from experimentally generated plumes. The comparison validates that the simulated plumes exhibit fine-scale qualitative and quantitative characteristics similar to experimental plumes. The ROS plume simulator facilitates future evaluations of environmental monitoring strategies by marine robots, and is made available for community use.

## 1. Introduction

The spatial and temporal evolution of a plume is affected by several factors of which advection, diffusion and weathering are dominant. Plume models have been developed to capture these influences in marine environments, such as models for algae blooms (Wong et al., 2007), subglacial melt water plumes (Carroll et al., 2015), and oil plumes. The oil plume modeling community alone has developed over fifty models, a handful of which are widely used (Yapa, 1996). These pollutant dispersion models can be broadly characterized into two main categories on the basis of the modeling method used, namely Eulerian dispersion models and particle-based Lagrangian models (Zannetti, 1990). The Eulerian models are based on the conservation of mass of a single pollutant species (Zannetti, 1990). Lagrangian environmental models consider plumes to be composed of particles or alternatively, filaments. Another classification of plume models can be based on the scale - in space and time - for which the model is capable of producing reliable predictions. This classification is discussed next.

### 1.1. Pollution Plume Models

A classification of the environmental models can be made on the basis of the spatial and temporal scales a given model is able to capture. In this section we group the Eulerian models and Lagrangian models into large-scale and fine-scale model categories for the purpose of this study. The goal of this exercise is to highlight the scarcity of existing models suitable for robotics domain. It is important to emphasize here that these are not new modeling techniques, rather a classification of existing models based on *spatiotemporal scales* they capture. This distinction is pivotal since robots typically operate at sub-meter spatial scales and sub-second time scales. Only models that capture plume structure at this scale are relevant for robotics studies. Majority of the recent research effort has focused on models which are not suitable for environmental monitoring studies using robots as highlighted by the existing literature in the sections below.

#### 1.1.1. Large-Scale Models

Large-scale models are time averaged, long term exposure capturing models. These models simulate plume spatiotemporal development at grid spacing of the order of 10–100 s of meters and at temporal steps of the order of minutes. These models are referred to, for the purpose of this work as *large-scale models*. Large-scale models are long term exposure capturing models, and are thus mainly “fate and transport” models that capture aggregate plume behavior, intended for activities such as assessing environmental damage as a result of oil spills. Both Eulerian and Lagrangian models have been used to model plume development in this domain. These models have been the subject of many studies by the oil spill modeling community (Yapa, 1996; Reed et al., 2000; Klemas, 2010; Cheng et al., 2011). French McCay proposed a time averaged Lagrangian environmental model simulator named SIMAP (McCay and French-McCay, 2003; French-McCay, 2004). This simulator was calibrated using results of field experiments (French-McCay et al., 2008). The National Oceanographic and Atmospheric Agency (NOAA) also developed a time averaged plume simulator called General NOAA Oil Modeling Environment (GNOME) (Beegle-Krause et al., 2001). It is also based on a Lagrangian model and was developed to aid planners and first responders for damage assessment in the event of an offshore oil spill. MEDSLICK-II is another time averaged Lagrangian model which is coupled with Eulerian circulation models (De Dominicis et al., 2013), and explores the reconstruction of concentration field from advection, diffusion and transformation processes. COZOIL (Howlett and Jayko, 1998) is another example of simulators in this category.

#### 1.1.2. Fine-Scale Models

In contrast to fate and transport models, another classification of plume models is fine-scale models. These are capable of predicting the instantaneous concentration field at fine spatial scales. These models capture plume development at sub-meter grid spacing, and sub-second time resolutions. We characterize these models as *fine-scale models*. Fine-scale models are designed for studies examining autonomous environmental monitoring problems (Jones, 1983). There are some existing models in this category that target robotics studies, mainly in the aerial plume modeling domain. Cabrita et. al. present PlumeSIM, a Stage/Player based plume simulator, and VirtualPlume, its Robot Operating System (ROS) implementation, for aerial plumes (Cabrita et al., 2010). Pashami et. al. present another filament based gas dispersion model integrated with OpenFOAM, which is a flow simulation software for the generation of realistic compressible and incompressible flow fields (Pashami et al., 2010). Vergassola et al. use turbulent transport of particles to model the plume as particles propogating with diffusivity (combining turbulent and molecular diffusion) and advected by a mean current or wind (Vergassola et al., 2007). This plume model is adopted by Ristic et al. in their work (Ristic et al., 2016). Jones provides empirical characterization of the instantaneous concentration profiles of aerial plumes (Jones, 1983). He observes that aerial plumes consist of short bursts of high concentration, interspersed with generally rather longer intervals of zero or near zero concentration, and refers to this phenomenon as intermittency. These bursts were also found to be sub-second in duration. Due to the short duration of these bursts, aerial plumes can most effectively be modeled using filament based Lagrangian models. Farrell et. al. have presented a fine-scale filament based Lagrangian environmental model for marine plumes (Farrell et al., 2002) using these observations for characteristic comparison. Most existing studies that capture the instantaneous concentration profiles of plumes are focused on aerial plumes and use the filament based Lagrangian model. In the next section, we provide an overview of research being conducted in the autonomous robotic environmental monitoring, highlighting the utility of a model that captures plume fine-scales.

### 1.2. Autonomous Robotic Environmental Monitoring

The use of robots for environmental monitoring is receiving increased research attention recently. A detailed review of work in this direction has been provided by Dunbabin and Marques (Dunbabin and Marques, 2012). They reviewed the research efforts being carried out in marine, aerial and terrestrial domains. Ishida et. al. provide a review focusing on the use of robots to monitor chemicals introduced in a fluid media (Ishida et al., 2012). The current research effort has mainly focused on source seeking, which involves the study of techniques and algorithms, designed to move a robot or group of robots to the source of a chemical plume. Odor tracking in aerial domain has been the focus of a few researcher efforts (Ishida et al., 1994;  1995; Hayes et al., 2002; Marques et al., 2006). A few of these studies have relied on small scale laboratory experiments for the verification of algorithms. Ishida has highlighted that these laboratory experiments create “significantly simplified environments” for algorithm verification, compared to real world conditions (Ishida, 2007).

Most of existing work in environmental monitoring using robots has focused on source seeking using either mapping based (Farrell et al., 2003a; Lilienthal and Duckett, 2004; Pang and Farrell, 2006; Ferri et al., 2011), behavior based (Bruemmer et al., 2002; Lilienthal and Duckett, 2003; Wei Li et al., 2006) or control based approaches (Clark and Fierro, 2005; Zhang et al., 2007; Hsieh et al., 2012). Farrell et. al. developed a hidden Markov model based approach for marine plumes (Farrell et al., 2003a) and evaluated the performance using an environmental model tuned to reproduce the characteristics of aerial plumes (Farrell et al., 2002). Li et. al. also used the models proposed by Farrell for the validation of their algorithm (Li and Sutton, 2007; Li et al., 2008). Pang and Farrell present a Bayesian inference model to generate a source-likelihood map based on real-time AUV observations (Pang and Farrell, 2006). They use the data from two of the four field campaigns described in (Farrell et al., 2003b; Farrell et al., 2005) for the verification of their proposed algorithm. Tian et. al. follow a strategy similar to Farrell, making use of an extension of the filament-based model proposed by Farrell, adding buoyancy terms in (Tian and Zhang, 2010; Tian et al., 2013; Tian et al., 2014). They include studies with tracer dye experimental evaluation of the biologically inspired behavior-based plume source localization techniques in (Tian et al., 2014). Vergassola et. al. present a macroscopic search strategy “Infotaxis” that maximizes the local rate of information gain (Vergassola et al., 2007). Following a “zigzagging” and “casting” path, the algorithm can be used for searching with sparse information in general and by olfactory robots in particular. A comparative study of cognitive search strategies to locate an emitting source using sparse non-zero sensor measurements is presented in (Ristic et al., 2016), where the sequential Monte Carlo method is used to estimate source parameters and the reward function for motion control. As highlighted by these studies, the autonomous monitoring of marine plumes is an important research topic. The methods used to verify autonomous plume monitoring algorithms have been based on models that relied on experimental data for aerial plumes. Pre-recorded experimental data has been used in certain studies (Pang and Farrell, 2006; Cabrita et al., 2010), which does not provide the diversity that would be required to reliably verify control algorithms. Repeated experimental verification can be both expensive and time consuming. This highlights the need for the development of a simulation model that captures the characteristics of marine plumes and at the same time, provides with sufficient varying conditions to reliably verify autonomous plume monitoring algorithms.

### 1.3. Contributions

The lack of experimental characterization of fine-scale characteristics of marine plumes motivated us to perform field experiments to quantify these characteristics (Fahad et al., 2017a; Fahad et al., 2017b). These experimental studies showed that while marine plumes exhibit characteristics qualitatively similar to aerial plumes, quantitatively they are disparate. The bursts of high concentration for instance, last much longer in the case of marine plumes. Overall observations suggested that the fine-scale ocean plume dynamics evolve over much longer time scales than the aerial plume structure previously studied and used. The more slowly varying characteristics can be attributed to slower advection velocities for marine environments and decreased diffusivity due to increased density in marine plumes as compared to aerial plumes. Thus existing instantaneous aerial plume models are insufficient to be realistically tuned to capture fine-scale characteristics of marine plumes.

In this paper, we present an Eulerian advection-diffusion model with time-varying flow field and intermittent source to capture the qualitative and quantitative fine-scale characteristics exhibited by experimental marine plumes. The model provides its user with sufficient parameters to vary different characteristics of the plumes to reflect the environmental conditions of interest. This work first summarize experimentally derived characteristics that were obtained from our field experiments (Fahad et al., 2017a; Fahad et al., 2017b). We then present the pollutant dispersion model with an intermittent source, together with an approximate solution method with affordable computational cost. The proposed model and solution method have been implemented in the ROS environment. A calibrated set of parameters is then presented that produces fine-scale characteristics that are qualitatively and quantitatively similar to those of experimentally generated marine plumes reported. Results of the comparison between the model and field experiment shows that the model exhibits fine-scale plume characteristics similar to those derived from experimentally generated marine plumes. We provide the conclusions drawn on the ability of this proposed model to capture realistic plume characteristics and the future work.

The model, implemented as a ROS simulator, has been made available for community use^1^. The simulator facilitates advanced robotic control algorithm testing with realistic ocean plume environments, reducing the need for expensive field testing during the development of new techniques for robotic plume monitoring. Comparing to our earlier work in (Fahad et al., 2015), that tests robot plume tracking controller in a robotic simulator, the scope and method are different in that: (1) a Lagrangian environmental model was used, while this paper uses an Eulerian partial differential equation (PDE) based advection dispersion model that is better suited for marine plumes; and (2) Field Robotics Vehicle Software (FVS) (Bingham et al., 2011) was used in (Fahad et al., 2015), while the current paper uses ROS thus benefits a much larger user community. The model gives its user the option to vary different parameters to vary characteristics of the plume. This would help in verifying any autonomous monitoring algorithm in a variety of varying conditions.

## 2. Fine-Scale Characteristics of Experimental Plumes

In this section, we summarize the qualitative and quantitative characteristics of experimentally generated plumes, obtained during a series of experiments conducted by our group (Fahad et al., 2017a; Fahad et al., 2017b). As mentioned in Section 1, the lack of experimental data for marine plumes prompted us to study these fine-scale characteristics for marine plumes. We conducted a series of field experiments by generating Rhodamine dye plumes in a near shore marine environment at Makai Research Pier in Oahu, Hawaii. A 20% Rhodamine solution mixed with seawater to achieve approximate neutral buoyancy, was introduced into the seawater using a metering pump at a flow rate of 5 ml/min. This pump was mounted on a floating platform moored at a fixed location. The solution was introduced through a small diameter hose mounted 0.1 m below the waterline. At this low flow rate the solution had negligible momentum when released into seawater. The resultant plumes were allowed to grow till their size was significant enough. These plumes were surveyed using unmanned surface vessels (USVs) equipped with fluorometer sensors. The sensors were installed 0.1 m below the waterline. Average winds of 5–15 mph were observed in the northeasterly direction consistent with the trade winds. The resulting plumes were observed to spread in the longitudinal direction at speeds between 0.03–0.1 m/s. Vertical spreading of the plumes was not measured. Three different types of surveys were performed to study the structure of the plumes. Time series of fluorometer sensors onboard the USVs were recorded during these surveys.

**Figure 1:**
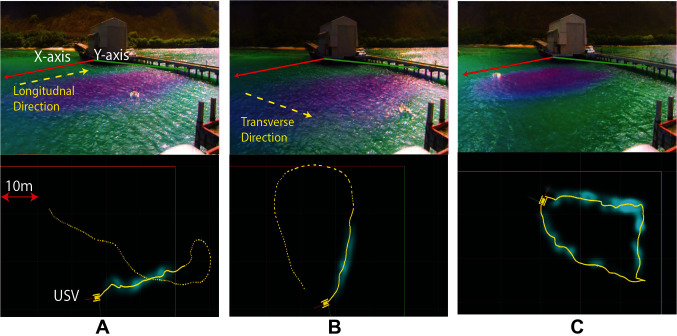
Field experimental snapshots (top panel) and recorded trajectories (bottom panel) visualized using ROS rviz: **(A)** Cross section survey in the longitudinal direction; **(B)** Cross section survey in the transverse direction; **(C)** Plume front survey.

- *Static surveys* were conducted by anchoring the USVs at fixed locations along the plume growth axis.
- *Cross section surveys* were performed by manually driving the USVs along the plume’s longitudinal and transverse axis of growth. The recorded USV trajectories in one of the cross section survey experiments are shown in Figure 1A and B for the longitudinal and transverse directions, respectively, where the top panels show camera snapshots and the bottom panels show ROS rviz visualization of the recorded trajectories.
- *Plume front surveys* were conducted by manually driving the USVs along the plume front. Plume front is defined to be the visual extent of the plume, i.e., the visual boundary where the plume is no longer visible to the human eye. The recorded trajectory of the USV during one of the plume front survey experiments is visualized in Figure 1C.

The static surveys were conducted during May 2015, and the results are presented in (Fahad et al., 2017a), while the cross section and plume front surveys were conducted during August 2015, and the results are presented in (Fahad et al., 2017b). Based on these experiments, the qualitative characteristics and the statistical parameters to quantify these qualitative characteristics are summarized below.

### 2.1. Qualitative Characteristics

Qualitatively, experimentally generated plumes exhibit intermittency, sinuous structure, coherence to flow field history and a distinct near source concentration profile which are explained in this section.

**Figure 2:**
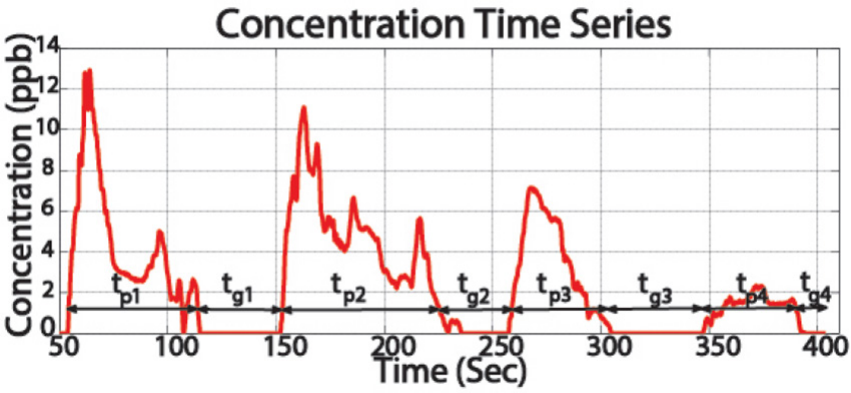
Time series of plume concentration measurement at a fixed spatial location showing bursts of high and low concentration.

**Figure 3:**
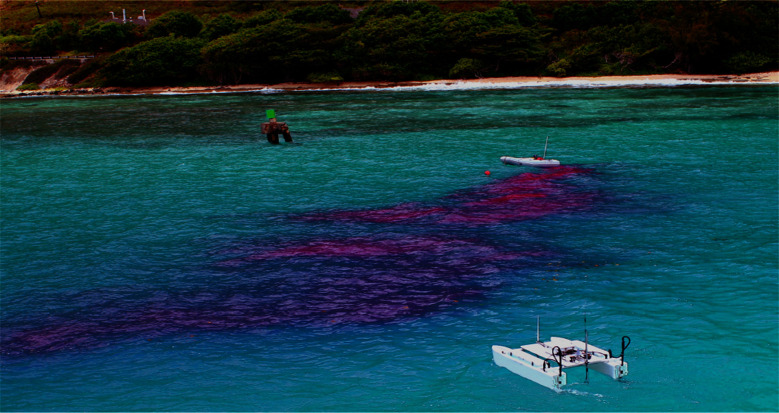
Snapshot of pollutant plume growth in one experiment showing sinuous and patchy behavior.

**Figure 4:**
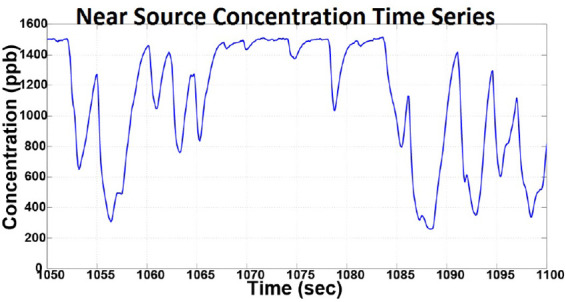
The concentration time series obtained by sampling the concentration near the plume source showing time varying concentration profile despite constant source release rate.

1. *Intermittency*: Intermittency is described as the cycles of high and low concentration, observed in a plume at a fixed location. This behavior is highlighted in the concentration time series plot shown in Figure 2, obtained during static plume surveys (Fahad et al., 2017a). The figure shows bursts of high concentration, of duration *t_pr_* and near zero concentrations of duration *t_gr_*. Here subscript *p* represents pulse time, and subscript *g* represents return time. It is important to highlight that pulse and return durations are not constant but random (Fahad et al., 2017a), which can be attributed to the complex intermixing of the pollutant and the fluid, and varying fluid flow.
2. *Sinuous structure*: Experiments showed that marine plumes follow a sinuous path. As shown in Figure 3, it can be seen that the plume follows a meandering path rather than a straight line which can be attributed to the time varying flow field experienced by the plume.
3. *Shape and flow field coherency*: Another important characteristic of an experimental plume is coherence of the plume shape and the temporal history of the flow field. The direction of advection of different sections of the plume should conform to the direction of the fluid flow during the initial growth of that section. In other words, the direction of growth of the different meandering sections of the plume is correlated to the direction of the fluid flow at the time of propogation of that section.
4. *Near source concentration profile*: The concentration profile near the source of the plume was studied by measuring the dye concentration 0.2 m downstream of the source. The plot of the measured time series is shown in Figure 4, which shows a time varying concentration profile, even when the flow rate from the source is constant. This varying concentration can be attributed to several factors, such as the vortex shedding generated due to the two mixing flows, large-scale and small scale eddies, and turbulence in the flow field.

### 2.2. Statistical Parameters

The fine-scale characteristics can be quantified using the set of statistical parameters detailed in this section. The time series obtained in field experiments were analyzed to calculate the metrics detailed here. These metrics provide a quantitative basis of comparison of fine-scale characteristics of simulated plumes and experimentally generated plumes presented in Section 5 of the paper.

1. Mean concentration $\bar{c}$ of a recorded time series at fixed and moving points in the plume.
2. Coefficient of variation (CV) is the ratio of the SD *σ_c_* of the time series and its mean value $\bar{c}$.
3. Peak to mean ratio (PMR) is the ratio of the maximum value of the time series and its mean value.
4. Intermittency is quantified as the probability of the measured concentration being lower than a certain threshold *τ*. The intermittency is calculated as
  $$\gamma =\frac{card\left(c<\tau \right)}{card\left(c\right)}$$
  where **card**() denotes the cardinality/size of the set.
5. Burst length *t_p_*, is the duration for which the measured concentration continuously stays above the threshold *τ*.
6. Burst return *t_g_*, is the duration for which the measured concentration continuously stays below the threshold *τ*.

Next, we present our model used to capture these characteristics exhibited by experimentally generated marine plumes.

## 3. Models and Solution Algorithms

We present in this section the plume dispersion model, flow field generation model, and plume source model for simulating marine plumes. The qualitative and quantitative characteristics of these simulated plumes are compared, and shown to be similar to experimentally generated plumes in Section 5. While explicit mathematical modeling of all the physical and chemical phenomena governing the development of a real world marine plume is overly complex (Moin and Kim, 1997; Shraiman and Siggia, 2000), our goal is to produce a more realistic, yet tractable simulation model for use by the robotics community in their control algorithm design studies. In this section, we provide the models and solution methods that produce an overall marine plume whose characteristics are similar to those exhibited by experimentally generated marine plumes.

### 3.1. Plume Dispersion Model

The spreading of a contaminant released into a fluid is affected by two dominant factors, namely, advection and turbulent diffusion (Socolofsky and Jirka, 2005). The PDE capturing these two effects and governing the spreading of the contaminant is given by (Socolofsky and Jirka, 2005)

(1) $$\frac{\partial c\left(\text{x},t\right)}{\partial t}+v_{x}\frac{\partial }{\partial x}c\left(\text{x},t\right)+v_{y}\frac{\partial }{\partial y}c\left(\text{x},t\right)=k_{x}\frac{\partial^{2}}{\partial x^{2}}c\left(\text{x},t\right)+k_{y}\frac{\partial^{2}}{\partial y^{2}}c\left(\text{x},t\right),$$

where *c *(x, *t*) is the pollutant concentration at position x = (*x, y*) and time *t*,

v = [*v_x_, v_y_*] is the flow field vector,

k = [*k_x_, k_y_*] is the turbulent diffusion coefficient vector.

In the limit k →(0,0), Eq. (1) is a purely advection model, and in the limit v →(0,0), it is a purely dispersive model.

There are several methods to solve Eq. (1) analytically for general initial and boundary conditions, when diffusion coefficients are constant and geometries are simple (Zoppou and Knight, 1997). In this work, the finite difference method (FDM) has been used to solve this equation. To solve Eq. (1) using FDM, the solution domain is discretized using a uniform rectangular grid of step size Δ*x* and Δ*y* in both directions. Here *l_x_* and *l_y_* are the total lengths of the environment along both axes, and *n_x_* and *n_y_* are the total number of grid point along *x*-axis and *y*-axis respectively. Grid points occur at the intersection of grid lines and are numbered as *i* = 1, 2, …, *n_x_* along *x*-axis and as *j* = 1, 2, …, *n_y_* along *y*-axis. The concentration value at the *n*-th time step at any (*i, j*)-*th* grid point is labeled as $c_{i,j}^{n}$. The simulation time *T_s_* is divided into *N* = *T_s_*/Δ*t* steps, where Δ*t* is duration of each time step represented by *n* = 1, 2, …, *N*. The concentration value at this point at (*n* + 1)-*th* time step is denoted by $c_{i,j}^{n+1}$ and approximated by

(2) $$c_{i,j}^{n+1}=c_{i,j}^{n}+f_{1}\left(x\right)+f_{2}\left(y\right)+f_{3}\left(x\right)+f_{4}\left(y\right)$$

where

$$f_{1}\left(x\right)=\frac{\left(v_{x}\left(i,j\right)\Delta t\right)}{\Delta x}\left[\left(v_{x}\left(i,j\right)>0\right)\left(c_{i+1,j}^{n}-c_{i,j}^{n}\right)+\left(v_{x}\left(i,j\right)<0\right)\left(c_{i,j}^{n}-c_{i-1,j}^{n}\right)\right]$$

$$f_{2}\left(y\right)=\frac{\left(v_{y}\left(i,j\right)\Delta t\right)}{\Delta y}\left[\left(v_{y}\left(i,j\right)>0\right)\left(c_{i,j+1}^{n}-c_{i,j}^{n}\right)+\left(v_{y}\left(i,j\right)<0\right)\left(c_{i,j}^{n}-c_{i,j-1}^{n}\right)\right]$$

$$f_{3}\left(x\right)=\frac{k_{x}\Delta t}{\Delta x^{2}}\left(c_{i+1,j}^{n}+c_{i-1,j}^{n}-2c_{i,j}^{n}\right)$$

$$f_{4}\left(y\right)=\frac{k_{y}\Delta t}{\Delta y^{2}}\left(c_{i,j+1}^{n}+c_{i,j-1}^{n}-2c_{i,j}^{n}\right)$$

Stability of the solution obtained by Eq. (2) is ensured by limiting the Courant number *C_r_* to

(3) $$C_{r}=|\frac{v_{x}\Delta t}{\Delta x}|+|\frac{v_{y}\Delta t}{\Delta y}|\leq 1.$$

### 3.2. Flow Field Model

The Navier Stokes equation is used to model the flow field in the environment and is given by

(4) $$\frac{\delta \text{v}}{\delta t}+\left(\text{v}\cdot \nabla \right)\text{v}=-\frac{1}{\rho }\nabla p+\nu \nabla^{2}\text{v}$$

where *ρ* is density of the fluid, *p* is the pressure field, and *ν* is the viscosity of the fluid for which the equations are solved. The incompressibility condition for a liquid medium is satisfied by

∇⋅v=0.

This model is then solved for each grid point of the simulation environment for given boundary conditions, giving the fluid velocity vector v at each point in the simulation environment. To simulate a time varying flow field, the boundary conditions are varied periodically and the flow field is updated accordingly.

### 3.3. Plume Source Model

Explicit modeling of a time varying source that captures all the effects that result in the near source concentration profile detailed in Secion 2.1 is computationally intractable (Moin and Kim, 1997; Shraiman and Siggia, 2000). In order to generate a plume that exhibits characteristics similar to experimental plumes, the plume source in this work has been modeled as intermittent. It cycles between a zero and non-zero concentration. The source concentration is given as

(5,6) $$c_{1}(t)=\left\{ \begin{array}{ll} c_{s}, & 0\leq t\leq T_{p}\\ 0, & T_{p}§lt;t§lt;T_{p}+T_{g} \end{array}\right.$$

where *c_s_* is the adjustable source concentration parameter, *T_p_* and *T_g_* are pulse time and gap time, respectively. The function *c*_1_ is repeated after *t* = *T_p_ +T_g_* for *m* times. The selection criterion of these parameters are given in Section 4.2. Next, we present the implementation of these models in the ROS simulator.

## 4. ROS Simulator

We present the implementation details of the models explained in Section 3 and also present the method of parameter selection to vary plume characteristics.

### 4.1. ROS Implementation

**Figure 5:**
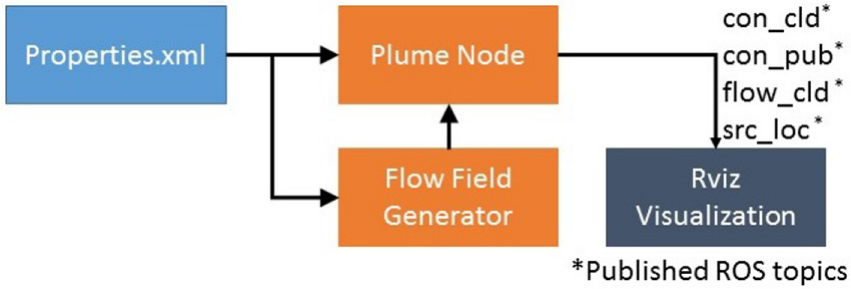
ROS Implementation block diagram.

The block diagram for the ROS implementation showing the major components is shown in Figure 5. Implementation details of each component are described below.

#### 4.1.1. Properties XML File

The *properties* extensible markup language (XML) file is an editable file and contains parameters that may be modified by the user to set up the simulation. The user can select the simulation environment size *l_x_, l_y_*, the grid step size Δ*x* and Δ*y* and total simulation time *T_s_*. The user can simulate multiple sources and can set each source’s location *x_s_, y_s_*, concentration *c_s_*, mean pulse time *T_p_* and mean gap time *T_g_*. The turbulent diffusion coefficients k can also be set here. The parameter *t_f_* is used to control the update rate of the flow field, and maximum flow velocities *v_xm_, v_ym_* can also be set.

#### 4.1.2. Flow Field Generator

The flow field generator implements the flow model detailed in Section 3.2. The flow field is updated by varying the boundary flow velocity values every *t_f_* seconds. The object reads the required parameters from the *properties* file and *v_x_, v_y_* and *p* are initialized. To obtain a time varying flow field, in our simulator implementation, the boundary conditions are modeled as uniformly distributed random variables over the range ±*v_xm_* and ±*v_ym_* and are varied every *t_f_* secs. The values for *v_x_* and *v_y_* are iteratively updated using Eq. (4), till a stable solution is achieved and this solution is saved. This process is repeated for a total of *T_s_/t_f_* iterations.

The flow field can be run real time or saved offline. Saving values of *v_x_* and *v_y_* offline for later use has three main advantages. First, the simulation is comparatively faster than if the flow field was calculated online during the simulation. Second, by using saved data, performance of different control algorithms can be evaluated using the same time varying flow field at each step. Third, the flow field can be generated and saved by a more complex modeling software such as OpenFOAM and then used with this simulator.

#### 4.1.3. Plume Node

The plume node contains the implementation of the environmental model presented in Section 3.1. The node executes using both the central processing unit (CPU) and the graphics processing unit (GPU) to speed up the solution of the model. The node sets up the simulation according to the parameters set by the user in the *properties* file. The time step Δ*t* is calculated using Eq. (3), based on the maximum flow velocity *v_xm_, v_ym_*. Four ROS topics, *con_cld, con_pub, flow_cld* and *src_loc* are published by the node. The user can subscribe to these topics in their simulation to display the plume and concentration values, concentration heatmap, the flow field and source locations.

#### 4.1.4. Rviz Visualization

**Figure 6:**
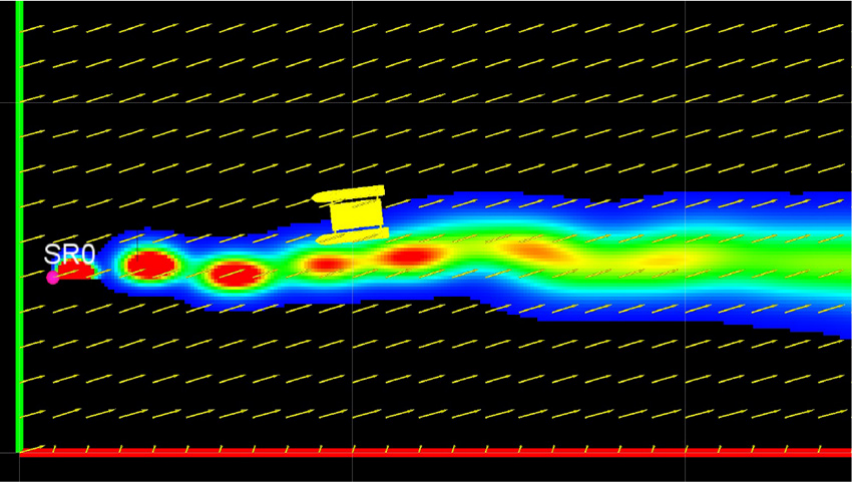
Rviz visualization of a plume generated by the plume simulator. A USV model is also shown integrated with the ROS plume simulator.

The plume generated by the plume node is displayed using the ROS visualization utility *rviz*. A snapshot from one of the generated plumes is shown in Figure 6. The concentration field is published as a point cloud of flat squares on the ROS topic *con_cld*. The flow field direction and magnitude is published as a marker array on topic *flow_cld*. The location of each simulated source is published as a marker array on the topic *src_loc*. These topics can be subscribed to in any other simulation to visualize the plume, the flow field and the source location. The plume concentration is visualized as a regular jet colormap, where red represents the highest concentration and blue represents the lowest concentration. Figure 6 also shows a USV model integrated with this simulator, which is part of FVS and serves two purposes. First, it was used to ensure that this simulator can be integrated with other ROS and non-ROS simulators. The second purpose was to perform plume front and cross section sampling detailed in Section 5.

### 4.2. Parameter Selection

In this section, we present the effect of parameters of the plume simulator on the fine-scale characteristics of the simulated plume, namely the turbulent diffusion coefficient and source gap time and the nominal ranges for adjustable parameters.

#### 4.2.1. Turbulent Diffusion Coefficient

**Figure 7:**
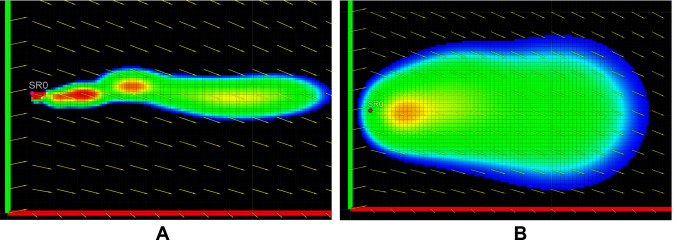
Plume growth with turbulent diffusion coefficient k = [*k_x_, k_y_*] = (10^–6^ m^2^/s,10^–6^ m^2^/s) shown in **(A)** and k = [*k_x_, k_y_*] = (0.01 m^2^/s, 0.01 m^2^/s) shown in **(B)**.

We present the effect of different turbulent diffusion coefficients on the growth of the plume. Figure 7A shows the plume growth for k = [*k_x_, k_y_*] = (10^–6^ m^2^/s,10^–6^ m^2^/s), while Figure 7B shows the plume growth with k = (0.01 m^2^/s,0.01 m^2^/s), at the same time instance. All other parameters and the flow field were kept the same between the two cases. Figure 7B shows more spread of the plume due to higher turbulent diffusion coefficient when compared to Figure 7A. It is also important to highlight the comparatively faster reduction in the visible intermittency for the higher k case, due to the improved intermixing.

#### 4.2.2. Gap Time

**Figure 8:**
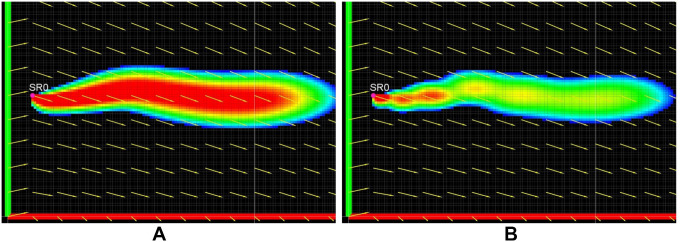
Plume intermittency by setting *T_g_* = 0 s in **(A)** and *T_g_* = 20 s in **(B)**.

The plume’s intermittency can be changed by changing the gap time *T_g_*. The growth of the plume for *T_g_* = 0 s is shown in Figure 8A showing no intermittency, while Figure 8B shows plume growth for *T_g_* = 20 s with visually observable intermittency. This highlights a proportional relationship of this parameter with intermittency.

#### 4.2.3. Parameter Ranges

**Figure 9:**
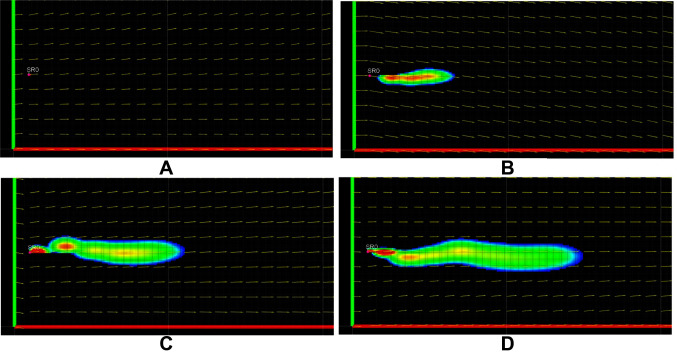
Snapshots of the plume during its growth at different time steps, T = 0 s in **(A)**, T = 60 s in **(B)**, T = 120 s in **(C)** and T = 180 s in **(D)**. Temporal parameters are set to *T_p_* = 2 s and *T_g_* = 10 s the diffusivity coefficient vector k = [*k_x_, k_y_*] is set to (10^–6^ m^2^/s,10^–6^ m^2^/s).

Explicit mathematical calculation of k, *T_g_* for the wide range of real field conditions is very difficult or impossible. The numerical values presented above quantify the expected range of these parameter to produce plume characteristic similar to experimentally generated plumes. These two parameters are important to adjust intermittency of the simulated plume and the values maybe be chosen to a higher or lower value, depending on the requirements, to produce varying plume characteristics. The simulated plume exhibits similar qualitative characteristics as an experimental plume. This can be verified by analyzing Figure 9, showing snapshots of the first 180 secs of the simulation. Intermittency can be observed in the figures in the from of visible patches of high and low concentration. The plume follows a sinuous path, which is clearly indicated by the meandering shape of the plume seen in Figure 9A to Figure 9D. The different sections of this meandering path are consistent with the direction of the flow during the development of that section of the plume.

A list of important parameters with nominal ranges is provided in Table 1. The value of *c_s_* can be increased to increase the downstream concentration of the plume and vice versa. The values of *T_g_* can be decreased to produce a less intermittent plume and vice versa. The value of *T_p_* can be increased to produce a less intermittent plume and vice versa. The value of k = [*k_x_, k_y_*] can be increased to reduce the intermittency of the plume and vice versa. The values of *v_xm_* and *v_ym_* can be increased to produce a more meandering plume and vice versa.

**Table 1:** Key parameters used in the simulator and nominal ranges.

Parameter	Name	Unit	Range
*c_s_*	Mean concentration of source	ppb	0.00–10,000.00
*T_p_*	Pulse time	sec	0.00–100.00
*T_g_*	Gap time	sec	0.00–100.00
k	Diffusion coefficient	m^2^/s	0.10–2.00 × 10^–6^
*v_xm_, v_ym_*	Maximum flow in X and Y direction	m/s	0.00–2.50

Next we present the comparison of this simulated plume with experimental marine plume.

## 5. Comparison with Experimental Characteristics

We perform a qualitative and quantitative analysis of the characteristics of plumes generated by this calibrated model with the characteristics of experimentally generated plumes. The simulation to collect the time series for comparison is carried out in an environment of size 60 × 20 m along the *x*-axis and *y*-axis respectively and grid resolution of 0.1 m. The total simulation time *T_s_* is 6000 s. The plume source is located at *x_s_*=(1 m, 5 m), source concentration *c_s_* is set to 1300 ppb, and temporal parameters are set to *T_p_* = 2 s and *T_g_* = 10 s respectively. The diffusivity coefficient vector k is empirically set to (10^–6^ m^2^/s,10^–6^ m^2^/s).

The simulated plume exhibits the same qualitative characteristics as an experimental plume. This can be verified by analyzing Figure 9, showing snapshots of the first 180 secs of the simulation. Intermittency can be observed in the figures in the from of visible patches of high and low concentration. The plume follows a sinuous path, which is clearly indicated by the meandering shape of the plume seen in Figure 9A to Figure 9D. The different sections of this meandering path are consistent with the direction of the flow during the development of that section of the plume. We would like to emphasize that the intention of this comparison is not to recreate the same time series as an experimental plume. The experimental data was collected over various plumes with varying environmental conditions, and getting a set of all parameters from a single plume is very challenging or impossible (see Section 6.3 on experimental challenges). We intend to show a general similarity in characteristics between simulated and experimental plumes.

### 5.1. Time Series of Stationary Sampling Point

We present comparisons of the fine-scale characteristics of the simulated plumes with the experimental plumes when measured from stationary sampling points. Corresponding to the static surveys referenced in Section 2, four static sampling points downstream of the simulated source were selected to collect the concentration time series. The location of these points were (6 m, 5 m), (11 m, 5 m), (16 m, 5 m) and (46 m, 5 m), making them 5 m, 10 m, 15 m and 45 m away from the source. The time series collected at these points are designated as S0-S3 and stored for calculating the statistical parameters. Only that part of the time series, when the plume has spatially grown enough to reach all sampling points was used.

#### 5.1.1. Amplitude Statistics

Comparison of amplitude statistics is shown in Table 2, where S0-S3 represent statistical data collected in our developed simulator, and P0-P3 represent field experimental data.

**Table 2:** Amplitude statistics at stationary sampling point.

Parameters	Experiments				Simulator			
	P0	P1	P2	P3	S0	S1	S2	S3
Distance to source (m)	6.70	22.50	34.90	51.30	5.00	10.00	15.00	45.00
Duration (min)	6.70	15.30	24.90	13.70	100.00	100.00	100.00	100.00
Mean: $\bar{c}$ (ppb)	3.11	11.24	9.40	14.85	28.80	18.44	14.42	9.38
Mean norm: $\bar{c}/c_{0}$	1.00	3.61	3.02	4.77	1.00	0.64	0.50	0.32
Std: *σ_c_*	3.30	6.40	6.50	4.10	25.47	13.68	10.47	6.12
CV: $\sigma_{c}/\bar{c}$	1.30	0.59	0.69	0.28	0.88	0.74	0.72	0.65
PMR	7.20	2.90	3.10	1.60	6.74	3.10	2.99	2.52
Intermittency	0.48	0.07	0.12	0.01	0.15	0.12	0.09	0.07

1. Mean Concentration $\bar{c}$ of S0-S3 shows a decreasing trend as the distance of the sampling points from the source increases. This is consistent with intuition and general established models. However, P0-P3 seem to imply an increasing mean concentration as distance from the source increases. This discrepancy can be attributed to several reasons such as varying surface advection from one experiment to the next, since each time series is obtained during separate experiments. The ocean waves move the USV around, resulting in the USV transitioning in and out of the plume. The narrow spatial profile of the plume closer to the source might also result in a lower mean value closer to the source, due to the USV moving in and out of the plume.
2. Coefficient of Variation (CV) $\sigma_{c}/\bar{c}$ shows a decreasing trend for P0-P3 as distance from the source increases and the value lies in the range 0.28 to 1.3. Results for S0-S3 also exhibit a general decreasing trend as distance from the source increases and the numerical values are also within the same range as experimental results. In this comparison, the metric CV has been used rather than the SD. CV is more useful in this case since the results compared are from different surveys and different sections of plumes with greatly varying mean values, and CV is a more appropriate metric for comparing variability in the time series in this situation (Stuart and Ord, 1994).
3. Peak to Mean Ratio (PMR) for P0-P3 shows a decreasing trend as the distance from the source increases. The same trend was observed in S0-S3. The average value for the experimental data calculated to be 3.7 is quantitatively similar to 3.84 for simulated plume data.
4. Intermittency for P0-P3 and S0-S3 show a general decreasing trend as a function of increasing distance from the source. The threshold *τ* for calculating intermittency was selected to be 1 ppb. The experimental values however decrease more rapidly compared to the simulator values.

#### 5.1.2. Temporal Statistics

This section presents the comparison of the temporal characteristics of S0-S3 and P0-P3. The temporal statistics of particular interest are the *burst length, t_p_* and *burst return, t_g_*. These parameters quantify the typical time scales at which the concentration is expected to vary. Analysis of P0-P3 shows that the pulse and gap times are in the order of 10 s of seconds to 100 s of seconds. Similar time scales are exhibited by S0-S3. The burst times for P0-P3 and S0-S3 are summarized in Table 3. Here we would like to emphasize that the times don’t have a one-to-one correspondence, rather we show similarity in the intermittency time scales.

**Table 3:** Burst length distribution statistics.

Parameters		Experiments				Simulator			
		P0	P1	P2	P3	S0	S1	S2	S3
Distance to source [m]		6.74	22.50	34.90	51.30	5.00	10.00	15.00	45.00
Burst length	*τ*	2.00	2.00	2.00	8.00	2.00	2.00	2.00	2.00
	Mean [s]	75.70	93.40	11.40	84.40	112.00	70.70	80.80	59.50
	Max [s]	95.90	222.00	319.00	418.00	347.00	156.00	175.00	67.00
Burst return	*τ*	2.00	2.00	2.00	8.00	2.00	2.00	2.00	2.00
	Mean [s]	20.40	5.40	2.80	5.90	47.90	69.90	53.00	14.00
	Max [s]	62.40	17.70	143.00	22.40	129.00	377.00	145.00	22.00

### 5.2. Dynamic Analysis

This section presents a comparison of the statistical parameters of the simulator generated plume when sampled from a dynamic platform. This was performed by integrating the FVS simulator’s USV model equipped with four sampling points with this plume simulator. The simulated plumes were then sampled by manually driving the USV model across the plume cross section and along its plume front. Statistical parameters of the logged time series are compared with experimental results. A snapshot of the simulator integrated with the USV model is shown in Figure 6.

#### 5.2.1. Cross Section Analysis

**Figure 10:**
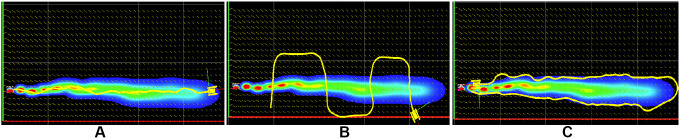
Simulated surveys with USV trajectories visualized in ROS rviz: **(A)** Cross section survey in the longitudinal direction; **(B)** Cross section survey in the transverse direction; **(C)** Plume front survey.

The cross sectional surveys were performed by driving the simulated USV along the longitudinal direction of the plume growth and along its transverse axis. The surveys SC1 and SC2 were conducted along the longitudinal direction of the experimental plume growth, one of which is visualized in Figure 10A. Similarly, surveys SC3-SC5 were conducted along the transverse axis of the plume growth. The representative trajectory for these surveys is shown in Figure 10B. The characteristics of the simulated plume along its cross section are compared here with the experimental plumes. The time series of experimental cross section surveys are labeled as C1-C6, while those obtained during the simulations are labeled as SC1-SC5. The statistical parameters for both are calculated and listed in Table 4. Comparison of the parameters show that the experimentally generated plumes and the simulated plumes exhibit quantitatively similar statistical parameters.

**Table 4:** Summary statistics of the concentration time series from plume cross section surveys.

Parameters	Experiments						Simulator				
	C1	C2	C3	C4	C5	C6	SC1	SC2	SC3	SC4	SC5
Mean (ppb)	16.83	16.93	7.23	11.30	6.44	14.68	11.70	5.80	6.75	5.66	7.16
Var (*σ*^2^)	75.02	26.73	30.37	42.45	16.19	34.39	112.82	28.95	41.2	29.28	37.14
STD (*σ*)	8.66	5.17	5.51	6.52	4.02	5.86	10.62	5.38	6.41	5.41	6.09
CV (*σ/μ*)	0.51	0.31	0.76	0.58	0.62	0.40	0.91	0.93	0.95	0.96	0.85
*c_max_* (ppb)	30.21	22.91	17.14	22.56	11.49	22.39	30.80	16.36	18.62	14.98	16.82
PMR (*c_max_/μ*)	1.80	1.35	2.37	2.00	1.78	1.53	2.63	2.82	2.76	2.65	2.35

#### 5.2.2. Plume Front Analysis

The plume front surveys were performed by driving the simulated USV along the visible boundary of the plume. The trajectory for one of the plume front surveys is shown in Figure 10C. Statistical parameters of the time series along the plume front are compared with the experimental plume front surveys in this section. The time series obtained from experiments are labeled as F1 and F2 and those obtained from simulator are labeled as SF1-SF4. Results of analysis of fine-scale parameters from both are detailed in Table 5. The table shows the qualitative similarity of the concentration time series of simulated plumes and experimental plumes. The main difference here being lower mean concentration values for the simulated case reflected in statistical parameters in Table 5, which are otherwise similar.

**Table 5:** Summary statistics of plume front surveys.

Parameters	Experiments		Simulator			
	F1	F2	SF1	SF2	SF3	SF4
Mean (ppb)	23.79	17.70	9.08	17.51	16.40	15.69
Var (*σ*^2^)	142.06	53.28	71.85	55.44	48.72	29.80
STD (*σ*)	11.92	7.29	8.48	7.44	6.98	5.46
CV (*σ/μ*)	0.50	0.41	0.93	0.42	0.42	0.35
*c_max_* (ppb)	48.50	39.83	44.60	35.69	39.97	42.74
PMR (*c_max_/μ*)	2.04	2.25	4.91	2.03	2.43	2.72

## 6. DISCUSSIONS

### 6.1. Result Comparison

The results presented in Section 5 demonstrate that the proposed simulation model can capture key characteristics of marine plumes, and exhibit fine-scale qualitative characteristics similar to experimental plumes. Due to the complexity of the plume propagation process in the presence of uncontrollable environmental conditions such as temperature, winds etc., we do not directly calibrate model parameters using experimental data. Our approach is to provide a general purpose ROS simulator, the parameters of which can be adjusted to vary the characteristics of the plume. A general guidance on how to adjust the simulator parameters is also provided. Our purpose is not to design the model for a specific experimental environment or scenario, but to enable the user to rapidly test their robot control algorithms in a wide range of conditions by adjusting parameters to different values following the guidelines in Section 4.2.

### 6.2. Scalability

The comparison of statistical parameters in Section 5 has been made on a domain size similar to the experimental environment roughly 60 m long. Although our qualitative results apply to ocean plumes of relatively small size, similar characteristic trends were reported for large size plumes in other studies (Jones, 1983; Farrell et al., 2002). Intermittency in our plume model exhibits inverse proportionality to increasing distance from the source, which is also exhibited by experimental aerial plumes (Jones, 1983). Meandering also becomes less discernible at increased distances for experimental plumes due to the increased cross sectional profile of the plume, which is consistent with larger size plumes. This evidence suggests that our proposed model and simulator may apply to ocean plumes of a larger size. It is worth mentioning that as robots operate at scales for which fine-scale characteristics are more appropriate than large-scale characteristics, the focus of our current paper is to provide a fine-scale plume simulator.

### 6.3. Experimental Challenges and Limitations

Conducting field experiments and collecting data in real marine environments is challenging. Characteristics of experimentally generated marine plumes depend on environmental conditions that cannot be controlled in a field setting. We observed that even for the duration of one survey mission, the direction of plume growth was not constant due to the combination of temperature changes and wind direction changes. It is very challenging to collect large volume of data quantifying plume characteristics in one testing due to the short time span of stable plumes. Also, the motion of the USV during the survey disturbs the plume and may change the structure of the plume. This also causes the experimental results to be not exactly the same as simulated plumes. Limitations in available resources for field experiments also contribute to limited amount of data collected. Thus, direct comparison of the simulated plume with a specific experimental plume is not possible. It is our intention to provide a general ROS simulator that produces fine-scale marine pollution plumes for robotics researchers to evaluate control algorithms without expensive field testings.

## 7. Conclusions

In this paper, we have presented a plume dispersion model calibrated to capture the characteristics of experimentally generated plumes. The modified model has been used to implement a marine plume simulator in ROS. The simulator provides the user with different parameters to select for different quantitative and qualitative behaviors of the generated marine plume. Sample plumes were generated in the simulator, and the fine-scale statistical characteristics of these simulated plumes were compared with those obtained from the experimentation results. The simulated marine plumes show similar qualitative and quantitative characteristics when compared to the experimental results. This calibrated model and the implemented marine plume simulator enables researchers to validate their environmental monitoring strategies using marine robots in a variety of conditions, reducing the need for expensive field experiments, a capability that is desirable to robotic control system researchers. Future work includes updating the current simulator to include a more realistic flow field model, that can simulate a more realistic flow field model. The current flow field model is unable to capture the effect of winds and local eddy current generation. Another avenue for improvement is to update the current two dimensional model to a three dimensional model.

## AUTHOR CONTRIBUTIONS

MF contributed design of the study and implementation of the simulator. All authors contributed conception of the study and discussions on the method. All authors contributed to manuscript revision, read and approved the submitted version.

## Conflict of Interest Statement

The authors declare that the research was conducted in the absence of any commercial or financial relationships that could be construed as a potential conflict of interest.

The reviewer, DP, and handling Editor declared their shared affiliation.

## Footnotes

1 https://www.github.com/mfahadrobotics/marineplumesim
